# RNA-Sequencing Reveals Heat Shock 70-kDa Protein 6 (HSPA6) as a Novel Thymoquinone-Upregulated Gene That Inhibits Growth, Migration, and Invasion of Triple-Negative Breast Cancer Cells

**DOI:** 10.3389/fonc.2021.667995

**Published:** 2021-05-04

**Authors:** Shiyi Shen, Chunli Wei, Junjiang Fu

**Affiliations:** Key Laboratory of Epigenetics and Oncology, Research Center for Preclinical Medicine, Southwest Medical University, Luzhou, China

**Keywords:** triple-negative breast cancer, thymoquinone, HSPA6, migration, invasion, RNA-seq

## Abstract

**Objective:**

Breast cancer has become the first highest incidence which surpasses lung cancer as the most commonly diagnosed cancer, and the second highest mortality among women worldwide. Thymoquinone (TQ) is a key component from black seed oil and has anti-cancer properties in a variety of tumors, including triple-negative breast cancer (TNBC).

**Methods:**

RNA-sequencing (RNA-seq) was conducted with and without TQ treatment in TNBC cell line BT-549. Gene Ontology (GO) function classification annotation, Kyoto Encyclopedia of Genes and Genomes (KEGG) pathway analyses for these genes were conducted. Western blot and semi-quantitative RT-PCR were used to verify the regulated gene. Functional assays by overexpression or knocking down were performed for HSPA6 and its mediator TQ for inhibiting growth, migration and invasion of TNBC cells. The regulatory mechanisms and prognosis for HSPA6 for breast cancer survival were conducted through bioinformatics and online databases.

**Results:**

As a result, a total of 141 downregulated and 28 upregulated genes were identified and 18 differentially expressed genes, which might be related to carcinomas, were obtained. Interestingly, GO and KEGG pathway showed their roles on anti-cancer and anti-virus. Further analysis found that the *HSPA6* gene was the high significantly upregulated gene, and showed to inhibit TNBC cell growth, migration and invasion. High expression of *HSPA6* was positively correlated with long overall survival (OS) in patients with breast cancer, indicating the tumor-suppressive roles for HSPA6. But DNA methylation of *HSPA6* may not be the regulatory mechanism for *HSPA6* mRNA upregulation in breast cancer tissues, although the mRNA levels of *HSPA6* were increased in these cancer tissues compared with normal tissues. Moreover, TQ enhanced the inhibitory effect of migration and invasion when HSPA6 was overexpressed; while HSPA6 was knocked down, TQ attenuated the effects of HSPA6-promoted migration and invasion, demonstrating a partially dependent manner through HSPA6 by TQ treatment.

**Conclusion:**

We have successfully identified a novel TQ-targeted gene *HSPA6*, which shows the inhibitory effects on growth, migration and invasion in TNBC cells. Therefore, identification of HSPA6 not only reveals a new TQ regulatory mechanism, but also provides a novel candidate gene for clinical management and treatment of breast cancer, particularly for TNBC.

## Introduction

As the malignant tumor, female breast cancer has become the first highest incidence which surpasses lung cancer as the most commonly diagnosed cancer, and the second highest mortality among women worldwide (1). In this year, breast cancer was estimated to reach 2.3 million new cases (11.7%), followed by cancers of lung (11.4%), colorectal (10.0%), prostate (7.3%), and stomach (5.6%) (1). The incidence for breast cancer in China is increasing year by year (2). The treatment of breast cancer includes radiotherapy, endocrine therapy, chemotherapy, biological targeted therapy and traditional Chinese medicine adjuvant therapy; but the efficacy still needs to be further improved to benefit the patients.

Thymoquinone (TQ) is a key component from black seed oil from traditional herb medicine and has anti-cancer properties in a variety of tumors (3, 4). Previous studies in our laboratory and others demonstrated that TQ has significant inhibitory effects on the migration and invasion on breast cancer cells, including triple-negative breast cancer (TNBC) (5–9). TNBC is the most aggressive and chemoresistant subtype in breast cancer, with a typical characterization of lack of receptor expressions of estrogen receptor (ER), progesterone receptor (PR), and human epidermal growth factor receptor 2 (HER2). The management for TNBC imposes an economic burden on the society and family and represents a main challenge for both patients and clinicians. New molecular targets and therapeutic reagents are required for improving TNBC patient prognosis and survival. The global regulatory effects and its targets by TQ in TNBC cells are still unknown. Thus, it is necessary to identify novel TQ-targeted genes for breast cancer, including TNBC.

Heat shock 70-kDa protein 6 (HSPA6) (OMIM: 140555), which is cytogenetically located on human chromosome 1q23.3, encodes a 70-kDa protein. HSPA6 was first identified by Leung et al. in 1990 as a stress-induced heat-shock gene (10). *HSPA6* and *HSPA7* were reported to share more than ninety percent nucleotide identity through their coding regions; but HSPA7 showed no protein-coding potential (11). Although HSPA6 was discovered three decades ago, the functional roles in cancer progression are unclear (12–14). Recently, HSPA6 was discovered to be dispensable for Withaferin A-mediated apoptosis/autophagy or migration inhibition of breast cancer (15). In this study, RNA-sequencing (RNA-seq) was performed and TQ-targeted gene *HSPA6* was successfully identified for TNBC inhibition functionally.

## Materials and Methods

### Reagents and Cell Culture

BT-549 and MDA-MB-231 cells, both are TNBC cell lines, and HeLa cells (cervical cancer cell line) were purchased from the American Type Culture Collection (Manassas, VA, USA). RPMI1640 and DMEM were purchased from Thermo Fisher Scientific (Waltham, MA, USA). The fetal bovine serum (FBS) was purchased from Pan Biotech (Bavaria, Germany). TQ was purchased from Sigma-Aldrich and dissolved in dimethyl sulfoxide (DMSO) (Corning, Manassas, VA, USA). For BT-549 cell culture, the RPMI1640 medium containing 10% of FBS, 0.023 U/ml of insulin was used. For MDA-MB-231 and HeLa culture, the DMEM medium containing 10% of FBS was used. Then we incubated the cells in an incubator at 37°C with a 5% CO_2_ air atmosphere.

### RNA Extraction, Library Preparation, and RNA-Sequencing

After BT549 cells were treated with TQ for 6 h, total RNA was extracted by TRIzol Reagent (Invitrogen, cat. No 15596026) as described previously (16, 17). DNA contamination should be removed by digestion with DNase I after RNA extraction. The concentration and quality of RNA was measured by detecting A260/A280 with Nanodrop^TM^ spectrophotometer (Thermo Fisher Scientific Inc. Waltham, MA, USA) and the integrity of RNA was verified with 1.5% agarose gel electrophoresis. Then Qubit 3.0 with Qubit^TM^ RNA Broad Range Assay Kit (Q10210, Life Technologies) was used to quantify the RNA. Preparation for stranded RNA-sequencing library was constructed with 2 μg of total RNA using KC-Digital^TM^ Stranded mRNA Library Prep Kit from Illumina (Catalog # DR08502, Wuhan Seqhealth Co. Ltd., China). Then, we got enriched and quantified library products with 200 to 500 bps in length for RNA-seq on Novaseq 6000 sequencer (PE150 model, Illumina), according to the instruction of NovaSeq 5000/6000 S2 Reagent Kit (cat #: 20012861, Illumina). Briefly, we firstly thawed the preconfigured sequencing by synthesis (SBS) reagent cartridge and the cluster generation reagent cartridge. The library and the SBS reagent cartridge were then mixed and denatured. Then, the library tubes were put into the thawed cluster generation reagent cartridge. Subsequently, we put the cluster generation reagent cartridge into the flow tank for running. Finally, we selected “sequence” in the software, set parameters and started running.

### RNA-Seq Data Analysis, GO and KEGG Analyses

After RNA-seq, we used Trimmomatic (version 0.36) to filter raw data, discarded the low-quality reads, and trimmed the reads contaminated by adaptor sequences to ensure the clean data were good enough to use for standard RNA-seq analysis (18). Then, they were mapped to the reference genome of *Homo_sapiens*. GRCh38 was from URL: ftp://ftp.ensembl.org/pub/release-87/fasta/homo_sapiens/dna/using STAR software. Reads mapped to the exon regions of each gene were counted by software of featureCounts (version 1.5.1, Bioconductor), and then Reads Per Kilobase per Million mapped reads (RPKM) was calculated. Using the edgeR package (version 3.12.1) (19), genes differentially expressed with and without TQ treatments were identified. To judge the significantly statistical significance of gene expression differences, a p value cutoff score of 0.05 and fold-change cutoff score of 2 were used. Gene ontology (GO) enrichment and Kyoto encyclopedia of genes and genomes (KEGG) pathway analyses were applied for differentially expressed genes, implemented with software for KOBAS (version: 2.1.1) with a p value cutoff score of 0.05 (20).

### Analysis of mRNA Expression by Semi-Quantitative RT-PCR

After extraction, 1 μg of total RNA was used to generate cDNA. The total volume of cDNA synthesis reaction system (reverse transcriptase/RT-PCR) is 10 μl, including 1 μl of dNTPs, 2 μl of 5 × RT buffer, 0.5 μl of random primer, 0.5 μl of RevTra Ace enzyme (which was purchased from TOYOBO company, China), 0.25 μl of RT-enhancer, 0.25 μl of super RI, approximate amount volume of RNase free water and 1 μg of total RNA were also added. The reactions were carried out in a Mastercycler gradient thermocyler (Eppendorf, Germany) as follows: 15 min at 37°C, 5 min at 50°C, 5 min at 98°C, final holding at 16°C. The reaction products were used as templates for semi-quantitative PCR (21). Primers 5’-tggacaaggcccagattcat-3’ and 5’-atcctctccacctcctcctt-3’ were used to measure *HSPA6* mRNA levels. Meanwhile, the 5’-acagtcagccgcatcttctt-3’ and 5’-ttgattttggagggatctcg-3’ were used to measure *GAPDH* mRNA level, which served as an internal control to show the difference of *HSPA6* mRNA level among the experimental groups. The semi-quantitative RT-PCR experiments were repeated three times.

### Western Blot Assays

The proteins were extracted with EBC lysis buffer, separated on polyacrylamide gel electrophoresis, and transferred to nitrocellulose membrane (BioRad, USA) (22). The membrane was then kept in 5% skim milk (1 × TBST) at room temperature for 1~2 h, shaken gently in primary antibody solution at 4℃ for 8~12 h, washed thrice with 1 × TBST, and then incubated with secondary antibody (tagged with HRP) for 2~4 h at room temperature. Finally, the membrane was washed thrice with 1 × TBST buffer. After chemiluminiscence reaction, the protein bands on the membrane were visualized by using a digital imaging system from BioRad Lab (Universal Hood II, Italy). The primary antibodies were anti-HSPA6 (Santa Cruz Biotechnology, Inc., CA, USA), anti-β-actin (Cell Signaling Technology, Inc., MA, USA), and anti-Flag (Sigma-Aldrich, Inc., MO, USA). The secondary antibodies, corresponding to primary antibodies, were anti-rabbit or anti-mouse (Cell Signaling Technology, Inc., MA, USA).

### HSPA6-Overexpressed and HSPA6-Knocking Down Cell Lines

To generate HSPA6-overexpressed cell lines, 500 ng of pcDNA3.1-C-(k)DYK-HSPA6 plasmid or pcDNA3.1-C-(k)DYK empty vector (Nanjing Genscript Inc., China) was transfected into HeLa cells, and 24 h after transfection, western blot was performed to test whether HSPA6 was successfully overexpressed. In BT-549 cells, knocking down of HSPA6 was achieved by transferring pHS-ASO-LW529, pHS-ASO-LW530 or pHS-ASO-LW531 (Beijing Syngentech Co., LTD., Beijing, China). Meanwhile, plasmid pHS-ASO-LW429 was transfected as a negative control. Three days after transfection, western blot was performed to test whether HSPA6 was successfully knocked down.

### Assays for Real Time CelI AnaIysis (RTCA)

We used a real time cell analyzer (xCELLigence RTCA DP, Roche, Germany) to analyze cell migration, invasion and growth index, which was reported previously (5, 22). A CIM plate was used for cell invasion/migration assays. The matrigel (cat #: 354277, BD Biosciences) was diluted in 1 × PBS at 1:40, and then added to its upper chamber and solidified in cell incubator at 37°C. After the glue was solidified (about 1~2 h), 10% serum supplemented medium was added to the lower chamber wells to induce cell invasion, and 100 μl of cell suspensions (total number of cells 5 × 10^3^) was added into the upper chamber. After installing the upper and lower boards, we started the experiment by setting up the program, and monitored the processes of cell invasion/migration every 15 min till the end of the experiments. About 7 h later, the experimental group was treated with TQ at a final concentration of 10 μmol/L. The cell migration test was similar to the invasion test, except that there was no matrigel in the superior chamber wells. The cell growth experiment was carried out with E-Plate. First, 50 μl of 10% serum supplemented medium was added to each well after the cells were digested and counted so that each 100 μl cell suspension containing 5×10^3^ cells was added to each well, and the experiment began. The methods of TQ treatment were same as the invasion and migration experiments. All experiments were repeated three times.

### Protein Expression Analysis

We utilized the data from Clinical Proteomic Tumor Analysis Consortium (CPTAC) in UALCAN (University of Alabama Cancer) database (23) (http://ualcan.path.uab.edu/cgi-bin/CPTAC-Result.pl?genenam=HSPA6&ctype=Breast) to analyze the HSPA6 protein expressions between normal tissues and breast invasive carcinoma (BRCA) tumor tissues.

### Methylation Analysis for HSPA6 Promoter

The methylation status of *HSPA6* promoter region in the tissues of BRCA patients from The Cancer Genome Atlas (TCGA)-BRCA was explored through the UALCAN database and the database of DNA methylation interactive visualization database (DNMIVD). The associations between the *HSPA6* expression and promoter methylation of *HSPA6* in the normal and BRCA tissues were conducted by the database of DNMIVD (http://119.3.41.228/dnmivd/query_gene/?gene=HSPA6&panel=Summary&cancer=BRCA) (24–26).

### Prognosis Analysis

The clinical data for breast cancers from GEO, EGA, or TCGA were used for an overall survival (OS) analysis (27). The two patient cohorts according to upper quantile expressions of *HSPA6* were compared using a Kaplan-Meier survival plot (https://kmplot.com/analysis/index.php?p=service) (27, 28). The gene name *HSPA6* was searched in the database website and the patients were split by median, with or without restriction to breast cancer subtypes.

## Results

### Results for Genes That Are Differentially Expressed by TQ Treatment in Breast Cancer Cells BT-549

**Figure 1:**
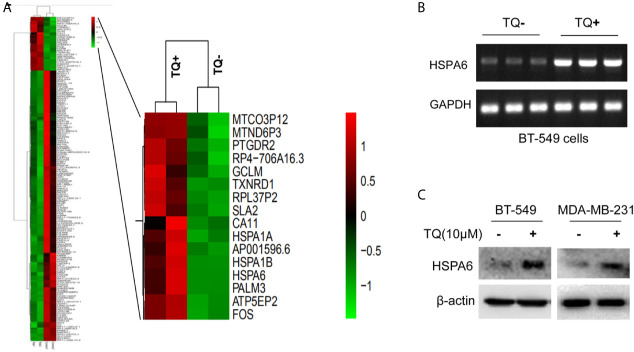
HSPA6 is a novel target by TQ regulation. **(A)** Clustering of differential genes from RNA-sequencing (RNA-seq) data with and without TQ treatments in TNBC BT-549 cells. Left panel, the heatmap of RNA-seq shows all significantly upregulated and downregulated genes after treated by TQ; right panel, the heatmap of RNA-seq shows part of significantly upregulated genes after treated by TQ. Red indicates highly expressed genes whereas green indicates lower expressed genes. A horizontal (X) axis presents different samples whereas a vertical (Y) axis presents the name of gene. The mRNA levels **(B)** and protein levels **(C)** for HSPA6 are increased by treatment with TQ for 6 h in the indicated breast cancer cell lines. The *GAPDH* and β-actin were set as internal controls for mRNA and protein respectively.

To identify globally affected target genes by TQ, RNA-seq was performed in TNBC cells BT-549 with or without TQ treatments. After RNA-seq, we have successfully identified a total of 141 downregulated and 28 upregulated genes (**Figure 1A** and **Supplementary Figure 1**, **Supplementary Table 1**, p<0.05). Then, GO enrichment and KEGG pathway analyses were performed to investigate the functions and pathways which are involved. Results for GO enrichment analysis of these differentially expressed genes in details are presented in **Supplementary Figure 2** and **Supplementary Tables 2, 3**, mainly in regulation of nucleotide-binding oligomerization domain containing 2 signaling pathway, positive regulation of tumor necrosis factor-mediated signaling pathway, protein refolding, cellular response to heat, viral life cycle, response to oxidative stress (GO up, **Supplementary Table 2**), negative regulation of myosin-light-chain-phosphatase activity, sister chromatid segregation, nuclear chromosome segregation, single-organism organelle organization, cytoskeleton, cell cycle (GO down, **Supplementary Table 3**), etc. Results for KEGG pathway analyses of differentially expressed genes are presented in **Supplementary Figure 3** and **Supplementary Tables 4, 5**, revealing that mainly in ribosome, longevity regulating pathway, legionellosis, estrogen signaling pathway, antigen processing and presentation (KEGG up, **Supplementary Table 4**), Fanconi anemia pathway, notch signaling pathway, Salmonella infection, pathways in cancer (KEGG down, **Supplementary Table 5**), etc.

### The Expression of HSPA6 Is Increased by TQ Treatment in Triple-Negative Breast Cancer Cells

From above differentially expressed genes, we found 18 differentially expressed genes, which might be closely related to carcinomas, either as oncogenes or tumor suppressor genes. After carefully analyzing, the *HSPA6* gene, as the highly significantly upregulated gene (**Figure 1A**, right panel) and involved into multiple pathways (**Supplementary Tables 2, 4**) by TQ treatment, was captured by us, and previous studies showed that this gene might be related to tumor repression (12). For further verification whether this gene had changes consistent with the results of RNA-seq in BT-549, we subsequently performed semi-quantitative RT-PCR and western blot. As expected, the obviously increased expression of mRNA level in BT-549 cells (**Figure 1B**) and protein level in both BT-549 and MDA-MB-231 cells (**Figure 1C**) were confirmed. Thus, *HSPA6* may be a novel TQ-targeted gene for our further study.

### HSPA6 Inhibits Cancer Cell Growth, Migration, and Invasion

**Figure 2:**
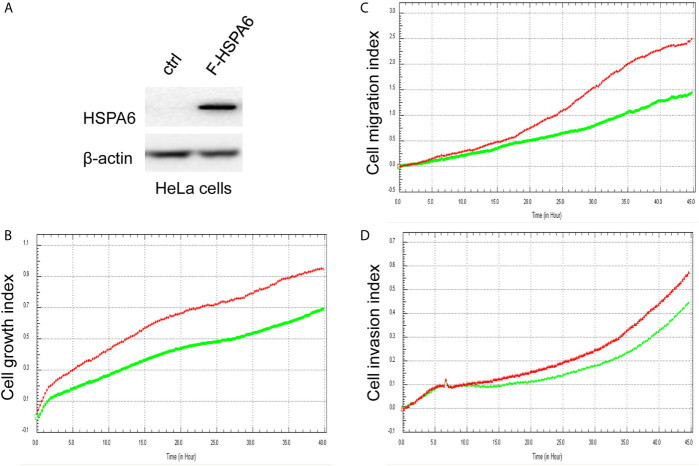
Overexpression of HSPA6 inhibits cancer cell growth, migration and invasion. **(A)** Overexpression of HSPA6 in HeLa cancer cell line. Lane “ctrl” indicates empty vector without HSPA6 expression as a control, whereas lane “F-HSPA6” indicates overexpressions of HSPA6 protein with western blot detected by Flag antibody. β-actin was set as an internal control for total protein loading. **(B)** Cell growth. **(C)** Cell migration. **(D)** Cell invasion. Red lines, controls; green lines, overexpressions of HSPA6.

**Figure 3:**
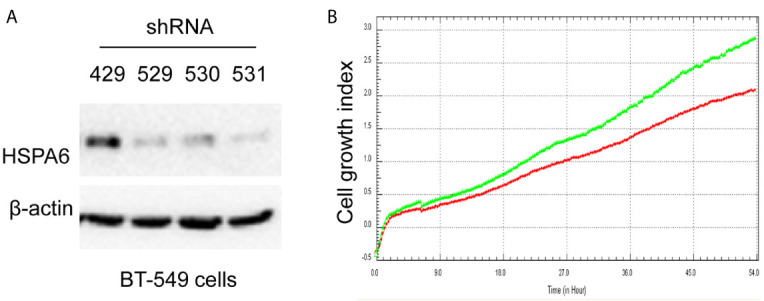
Knocking down HSPA6 promotes cancer cell growth. **(A)** Knocking down HSPA6 in TNBC cell line BT-549. Clones 529, 530, and 531 show the efficiency for knocking down of HSPA6, and clone 531 shows more efficiency; whereas clone 429 shows the empty vector control without knocking down. **(B)** Cell growth. Red lines, controls; green lines, knocking down HSPA6.

Based on the above experimental data, we identified HSPA6 as one of the target genes of TQ. In order to further verify the inhibitory effect of HSPA6 on cancer cell growth, we performed HSPA6 overexpression on HeLa cells with undetectable endogenous HSPA6. To do so, we transfected HSPA6 plasmid into HeLa cells and western blot was performed to check whether it was successfully expressed. **Figure 2A** shows that empty vector in HeLa cells did not express HSPA6, and the HSPA6 plasmid with Flag tag was successfully expressed. On the basis of this successful experiment, we further checked the effect of HSPA6 overexpression on cell growth, migration and invasion by RTCA assays. As presented in **Figures 2B–D**, HSPA6 did inhibit the cell growth, migration and invasion (**Figures 2B–D**). On the other hand, knocking down of HSPA6 in breast cancer cells BT-549 with highly endogenous expression was performed by using three shRNA plasmids. **Figure 3A** shows that HSPA6 was successfully silenced by all three shRNA plasmids, indicating plasmid 531 with more efficiency. Further RTCA assays revealed that the growth curve of BT-549 cells was significantly higher than that of the control group (**Figure 3B**). In addition, this inhibitory effect of HSPA6 may not be affected throughout cell cycle (**Supplementary Figure 4**).

**Figure 4:**
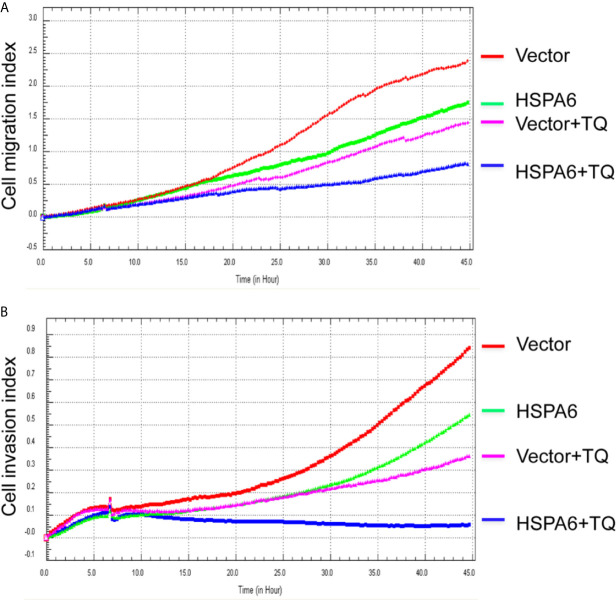
TQ enhances the inhibitory effect of cell migration and invasion when overexpression of HSPA6, demonstrating a partially dependent manner on HSPA6. **(A)** Cell migration. **(B)** Cell invasion. The efficiency for overexpression of HSPA6 was shown in **Figure 2A**. “vector” indicates the empty vector without HSPA6 expression, whereas “HSPA6” indicates overexpression of HSPA6.

**Figure 5:**
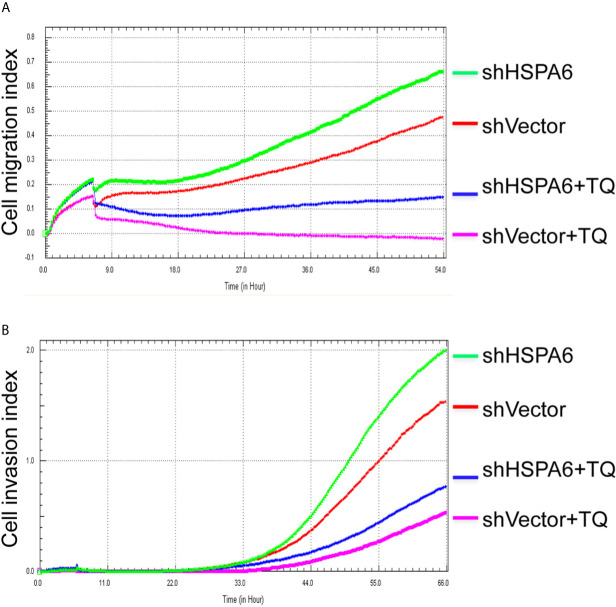
TQ attenuates the inhibitory effect of cell migration and invasion for HSPA6 when knocking down of HSPA6. **(A)** Cell migration. **(B)** Cell invasion. The efficiency for knocking down of HSPA6 was shown in **Figure 3A**. “shHSPA6” indicates knocking down of HSPA6 for clone 531, and “shVector” indicates the empty vector as a control without knocking down.

Then, we’d like to further ask whether HSPA6 inhibits cancer cell migration and invasion, the results by RTCA assay found that HSPA6 inhibited the migration (**Figure 4A**, red line *vs*. green line) and invasion (**Figure 4B**, red line *vs*. green line) when HSPA6 was overexpressed; while knocking down of HSPA6 promoted the migration (**Figure 5A**, red line *vs*. green line) and invasion (**Figure 5B**, red line *vs*. green line) in TNBC BT-549 cells.

Taken together, these studies strongly demonstrated the inhibitory effects of HSPA6 on tumor cell growth, migration and invasion.

### TQ Enhances the Inhibitory Effects of Cell Migration and Invasion When HSPA6 Was Overexpressed, While Knocking Down Attenuates the Effects

It has been reported that TQ inhibits breast cancer cell migration and invasion (5, 8), and further study here reveals that TQ upregulates HSPA6 expression. With these regards, by overexpression or knocking down of HSPA6 and then assays of cell migration and invasion were performed by RTCA. And the results found that TQ enhanced the inhibitory effect of cancer cell migration (**Figure 4A**, blue line *vs*. pink line) and invasion (**Figure 4B**, blue line *vs*. pink line) when HSPA6 was overexpressed; when knocking down HSPA6, TQ attenuated the inhibitory effects of cell migration (**Figure 5A**, red line *vs*. green line) and invasion (**Figure 5B**, red line *vs*. green line) of HSPA6-promoted, thus demonstrating a partially dependent manner through HSPA6 by TQ treatment.

### The Mechanism for Regulation of HSPA6 Expression in Breast Cancer Tissues

**Figure 6:**
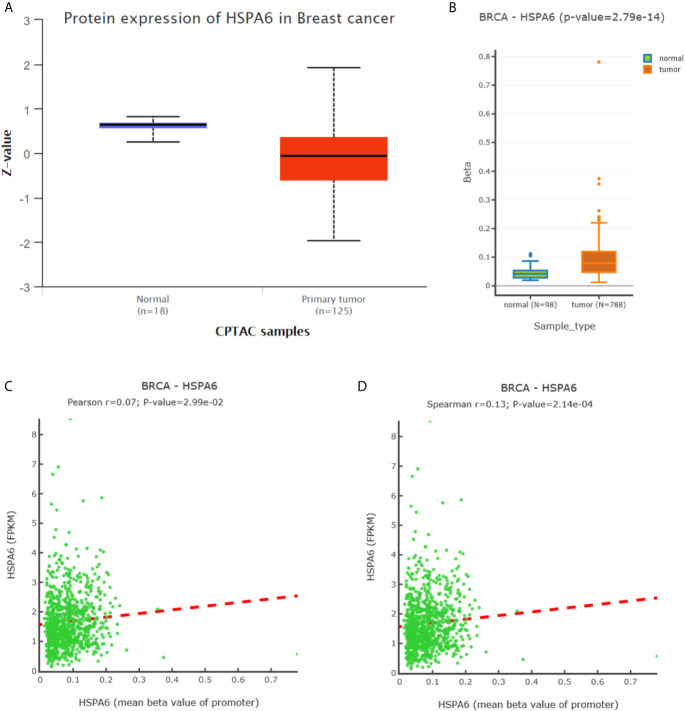
Expression and DNA methylation of HSPA6 in breast cancer tumor tissues. **(A)** HSPA6 protein levels in normal samples and breast cancer tumors (CPTAC samples) (p<0.01). Z values represent standard deviations from the median across samples in BRCA. **(B)** Boxplots of DNA methylation for *HSPA6* in BRCA (promoter region) (p<0.01). **(C)** Scatter plots of methylation-expression Pearson correlation for *HSPA6* in BRCA. **(D)** Scatter plots of methylation-expression Spearman correlation for *HSPA6* in BRCA. CPTAC, Clinical Proteomic Tumor Analysis Consortium. BRCA, breast invasive carcinoma. Horizontal (X) axes present *HSPA6* promoter methylation values whereas vertical (Y) axes present the HSPA6 mRNA expression level (FPKM). FPKM, Fragments Per Kilobase of exon model per Million mapped fragments.

To further investigate the HSPA6 expressions and its clinical significance in breast cancer patients, we thus utilized the data from CPTAC, and results showed that the HSPA6 protein expressions were decreased in breast cancer tissues compared with normal tissues (**Figure 6A**). However, the mRNA levels of *HSPA6* were increased in breast cancer tissues compared with normal tissues (data not shown). The mechanistic study by *HSPA6* promoter analysis indicated that the promoter regions of *HSPA6* in BRCA samples were increased in cancer tissues compared with matched normal tissues (**Figure 6B**), indicating that DNA methylation of *HSPA6* may not be the regulatory mechanism for *HSPA6* mRNA upregulation in those breast cancer tissues. And promoter methylation and HSPA6 expression in BRCA were also positively correlated (**Figures 6C, D**).

### High Expression of HSPA6 Is Positively Correlated With Long Overall Survival in Both All Subtypes of Breast Cancer Patients and TNBC Patients

**Figure 7:**
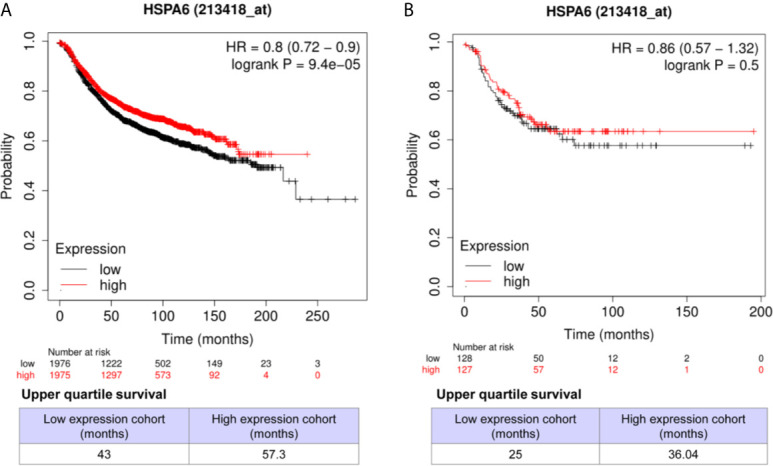
High expression of *HSPA6* is correlated with long overall survival for breast cancer patients in clinic. **(A)** All subtypes of breast cancer patients. **(B)** TNBC patients. The breast cancer patient samples are split into two groups according to the indicated upper quantile expressions of *HSPA6* from dataset of 213418_at. The HR with 95% confidence intervals and logrank p value were calculated. The logrank p value <=0.5 was set as a difference and logrank p value <=0.01 was set as a significant difference. HR, hazard ratio.

Through analyzing the clinical data of breast cancer (samples 213418_at) from Kaplan-Meier Plotter database, we found that high expression of HSPA6 was positively correlated with long overall survival (OS) in patients with both all subtypes of breast cancer (low expression cohort *vs*. high expression cohort for upper quantile expressions of HSPA6 were 43 months *vs*. 57.3 months) (**Figure 7A**, HR=0.8, 95% CI: 0.72~0.9) and TNBC (low expression cohort *vs*. high expression cohort for upper quantile expressions of HSPA6 were 25 months *vs*. 36.04 months) (**Figure 7B**, HR=0.86, 95% CI: 0.57~1.32), indicating the tumor-suppressive roles for HSPA6 in breast cancer. In another set of samples (117_at) from Kaplan-Meier Plotter database, similar results were also obtained (data not shown). But we should point out, p value was large than 0.05 in TNBC patients, it may be due to small sample numbers. Nevertheless HSPA6 can serve as a prognostic marker for breast cancer.

## Discussion

In order to identify target genes/pathways globally affected by TQ, RNA-seq was performed in TNBC cells BT-549, a total of 141 downregulated and 28 upregulated genes were found. GO function classification annotation showed mainly in protein refolding, cellular response to heat, nuclear chromosome segregation, sister chromatid segregation, microtubule cytoskeleton, chromosome segregation, single-organism organelle organization, cell cycle, viral life cycle, response to oxidative stress, etc.; KEGG pathway revealed mainly in Fanconi anemia pathway, Salmonella infection, pathways in cancer, or ribosome, longevity regulating pathway, legionellosis, estrogen signaling pathway, antigen processing and presentation, etc. Genes demonstrating in pathways of cancer and in viral life cycle indicate that TQ has roles for both anti-cancers and anti-viruses. Interestingly, recent studies found that TQ might have inhibitory potential against severe acute respiratory syndrome coronavirus 2 (SARS-CoV-2) protease (29), particularly for cancer patients (30). As we know, novel virus SARS-CoV-2 causes coronavirus disease 2019 (COVID-19), and the World Health Organization (WHO) declared COVID-19 as a global pandemic as earlier of March 11, 2020 (31–33). As of the March 22, 2021, the total confirmed cases are 123,719,955, and death cases are 2,724,465 worldwide from the report of Johns Hopkins University (https://coronavirus.jhu.edu/).

From differentially expressed genes, we found the *HSPA6* gene was the high significantly upregulated gene by TQ treatment in BT-549 TNBC cells, and showed that HSPA6 inhibited TNBC cell growth, migration and invasion *via* overexpression and knocking down assays. Through analyzing the clinical data of breast cancer by Kaplan-Meier Plotter, we found that high expression of HSPA6 was positively correlated with long OS in patients with both all subtypes of breast cancer and TNBC, indicating the tumor-suppressive roles for HSPA6. Thus, the data through bioinformatics analysis of multiple databases support the inhibitory effect of HSPA6 on breast cancer. Then, further mechanistic study showed that, although the mRNA levels of *HSPA6* were increased in breast cancer tissues compared with matched normal tissues, the promoter regions of *HSPA6* in BRCA samples were increased in cancer tissues compared with matched normal tissues, indicating that DNA methylation of *HSPA6* may not be the regulatory mechanism for *HSPA6* mRNA upregulation in those breast cancer tissues. And correlation for promoter methylation and *HSPA6* expression in BRCA was positively related. These data suggest that, in addition to heat stress, other mechanisms, such as small molecules for example TQ, should be involved in HSPA6 upregulation. Thus, these studies strongly demonstrated the inhibitory effects of HSPA6 on tumor cell growth, migration and invasion.

TQ has been reported to inhibit breast cancer cell migration and invasion and epithelial-mesenchymal transition (EMT) markers (5, 9, 34), and our RNA-seq data further revealed that TQ upregulates HSPA6 expression. With these regards, by overexpression or knocking down of HSPA6, the inhibitory roles of cell migration and invasion by TQ were performed, and we found that TQ enhanced the inhibitory effects of cancer cell migration and invasion when HSPA6 was overexpressed; while knocking down, TQ attenuated the inhibitory effect of growth, migration and invasion of HSPA6-promoted, thus demonstrating a partially dependent manner through HSPA6 by TQ. Altogether, identification of TQ-targeted HSPA6 not only reveals a new TQ regulatory mechanism, but also provides a novel candidate target for clinical management and treatment of breast cancer, particularly for TNBC upon TQ.

## Conclusions

By RNA-seq, we have successfully identified a novel TQ-targeted gene *HSPA6*, which showed the inhibitory effects on growth, migration and invasion in TNBC cells. The *HSPA6* promoter DNA methylation may not be the cause for *HSPA6* mRNA upregulation; other mechanism should be involved. Overexpression or knocking down of HSPA6 demonstrates a partially dependent manner through HSPA6 by TQ for HSPA6 inhibitory effects on TNBC cell growth, migration and invasion. Altogether, identification of HSPA6 will provide a novel candidate target for clinical management and treatment of breast cancer, particularly for TNBC on TQ.

## Data Availability Statement

The original contributions presented in the study are included in the article/**Supplementary Material**. Further inquiries can be directed to the corresponding author.

## Author Contributions

JF: designed and supervised the project. SS and CW: experimental studies. JF and SS: bioinformatics analysis. JF: wrote and edited the manuscript. All authors contributed, read and approved the submitted version.

## Funding

This work was supported by the National Natural Science Foundation of China (grants 81672887, 82073263, 81172049) and the Joint Research Foundation of Luzhou City and Southwest Medical University (2018LZXNYD-YL01).

## Conflict of Interest

The authors declare that the research was conducted in the absence of any commercial or financial relationships that could be construed as a potential conflict of interest.
