# Supplementary material for: RNA-Sequencing Reveals Heat Shock 70-kDa Protein 6 (HSPA6) as a Novel Thymoquinone-Upregulated Gene That Inhibits Growth, Migration, and Invasion of Triple-Negative Breast Cancer Cells
Source: Front Oncol. 2021 May 4;11:667995. doi: 10.3389/fonc.2021.667995 (PMC8129564; doi:10.3389/fonc.2021.667995)
Supplement: Supplementary file 1 [file DataSheet_1.docx]

Supplementary Material

# Supplemental Methods

**List of abbreviations**

| **Abbreviation** | **Meaning** |
| --- | --- |
| **APC** | Allophycocyanin |
| **BD** | Becton, Dickinson and Company |
| **CAPRI** | Ciclosporin to reduce reperfusion injury in primary PCI |
| **CCR7** | CD197 |
| **CD** | Cluster of differentiation |
| **cMRI** | Cardiac magnetic resonance imaging |
| **FITC** | Fluorescein isothiocyanate |
| **FMO** | Fluorescence minus one |
| **MFI** | Mean fluorescence intensity |
| **PCI** | Percutaneous coronary intervention |
| **PE** | Phycoerythrin |
| **STEMI** | ST-segment elevation myocardial infarction |
| **TCM** | Central memory T-cell |
| **TEM** | Effector memory T-cell |
| **TEMRA** | CD45RA+ effector memory T-cell |
| **TIMI** | Thrombolysis in myocardial infarction |
| **TNaïve** | Naïve T-cell |

***Populations***

We analysed several cohorts of patients in this study, shown schematically in **Figure 1**. To investigate long-term mortality in a large cohort, we retrospectively analysed 4874 consecutive patients presenting with STEMI, who were treated with pPCI at the Freeman Hospital, Newcastle between 2008 and 2015 (**Supplemental Table 3**). Admission lymphocyte counts were taken as pre-reperfusion values, as all patients have blood tests prior to reperfusion. Mortality was ascertained from the Office of National Statistics on 20^th^ June 2016. As this is a cohort of all pPCI patients in our centre over a long period, STEMI patients recruited to other cohorts analysed in this manuscript will usually also be included in this retrospective analysis, with the exception of the *TACTIC* trial (recruitment started after 2015).

The principle cohort for detailed lymphocyte/ CX_3_CR1 analysis was 52 STEMI patients who we recruited for the *CAPRI* study in 2015/16 (**Table 1**). These patients were treated with pPCI and standard care, and were randomised to receive an intravenous bolus of either ciclosporin (n=26) or placebo (n=26) immediately prior to reperfusion, and had arterial blood samples taken prior to reperfusion and at 5, 15, 30 and 90 minutes post-reperfusion. Venous blood was obtained at 24 hours after reperfusion, and at 2 weeks in 14 patients. Blood samples were analysed with flo cytometry as described below. Importantly, no difference in T-lymphocyte characteristics were observed between patients in the treatment and control arm of this study.^14^ 51 of these patients also underwent cardiac MRI (cMRI).

A further 50 STEMI patients, recruited for an observational study by our group led by Stephen Boag (data from which was published in part in the *Journal of Clinical Investigation* in 2015),^5^ underwent cMRI and these results were combined with *CAPRI* to give 101 acute post-STEMI cMRIs (**Supplemental Table 5**). A cohort of 19 STEMI patients receiving pPCI and 10 healthy controls, recruited for a separate non-interventional study, was used to establish baseline CX_3_CR1 expression in healthy controls, and analyse co-expression of CD27 and CD57 with CX_3_CR1 (**Supplemental Table 6**). Co-expression of CX_3_CR1 and CD28 was measured in a final cohort of 25 STEMI and NSTEMI patients, recruited for the *TACTIC* trial. Ex-vivo experiments were conducted using samples from healthy volunteers of unknown CMV serostatus.

***Inclusion and exclusion***

All STEMI patients in these populations had to be at least 18 years of age, presenting within 6 hours of onset of chest pain and ST-elevation. At admission coronary angiography, the culprit artery had to be a major vessel with diameter at least 3mm and have TIMI flow grade 0-1.

The datasets differed in their exclusion criteria, with *CAPRI* being the most stringent and the n=19 and n=25 cohorts being the least. In these last two cohorts, patients were excluded only for being clinically unstable, or having had a previous MI or thrombolytic therapy.

In the dataset collected by Stephen Boag, exclusion criteria were previous coronary artery bypass graft, active malignancy or infection, chronic inflammatory disease requiring immunosuppressive therapy, collateral circulation to the infarcted area and contraindications to cardiac MRI such as implanted devices, claustrophobia, etc.

All the above restrictions applied to *CAPRI* but, as the study also involved giving an investigational medical product (ciclosporin) and measuring one-year mortality, there were stricter criteria for the patient’s current medication and general health. Additional exclusion criteria were known sensitivity to ciclosporin or to egg, peanut or soya-bean proteins, known liver or kidney insufficiency, uncontrolled hypertension (>180/110mmHg), treatment with any compound which may modify blood concentration of ciclosporin, female patients either currently pregnant or of child-bearing age with no current contraceptive use, use of investigational study drug within previous 30 days and life expectancy of less than one year due to non-cardiac illness.

***PCI procedures***

PCI was performed according to standard guidelines, with initial loading with two antiplatelet drugs and heparin. Thrombus aspiration and glycoprotein IIb/IIIa inhibition were used according to the operator’s discretion. Zotarolimus or everolimus-eluting stents were used.

***Determining CMV status***

Blood was taken from the coronary arteries before reperfusion and analysed by enzyme-linked immunosorbent assay techniques in the clinical sciences laboratory at the Freeman Hospital, Newcastle-upon Tyne, UK.

***Flow cytometry assays***

Three different assays were used for flow cytometric analysis in this study; TruCount, 6-colour and 8-colour. Multicolour assays involved the use of fluorescence minus one (FMO) control samples; this enabled accurate gating of the positive population. Upon arrival, samples were immediately placed on a Stuart® roller mixer SRT6, ensuring sufficient mixing, and kept in the dark at room temperature. Incubation phases for all assays were carried out in the dark at room temperature.

*TruCount*

The absolute cell count of the various T-cell subpopulations was measured using a TruCount assay. 50μL fresh blood aliquots from each time point was added to their corresponding BD TruCount tubes (340334, BD Biosciences) using an eLINE Electronic 1-channel Pipette (15005787, Sartorius). Extra caution was exercised in ensuring that the tip is clean and rid of any blood as this could affect the absolute count of the cell populations. 10μL of BD Multitest 6-Color TBNK (337166, BD Biosciences) was deposited on the side of the tube, ensuring no contact was made with the sample. The BD Multitest 6-Color TBNK consisted of CD3 Fluorescein isothiocyanate (FITC), CD16 Phycoerythrin (PE), CD56-PE, CD45-Per-Cy5.5, CD4-PE-Cy7, CD19 Allophycocyanin (APC) and CD8-APC-Cy7 in buffer with 0.1% sodium azide. After vortexing the test sample using a Vortex Genie 2 (Scientific industries), the mixture was incubated before achieving red blood cell lysis by adding 1.5mL of fresh lysis buffer prepared using BD Pharm Lyse Lysing Buffer (10x Conc. 5075567, BD Biosciences) and Gibco distilled water (15230-147, Life technologies). After another period of incubation, the samples were run through a BD FACSCanto II Flow Cytometer (339473, BD Biosciences) using the BD FACSCanto acquisition software, which was set up using BD FACS 7-color Setup Beads (335775, BD Biosciences) and BD FACS Setup Bead Diluent (336565, BD Biosciences).

*6-colour assay*

Four sub-populations of CD4^+^ and CD8^+^ T-cells were investigated: T_Naive_, T_CM_, T_EM_ and T_EMRA_ , as previously described [PMID:10537110]. 50μL fresh blood aliquots from every time point was added to their corresponding labelled 5mL Falcon tubes. A cocktail of antibodies, detailed in **Supplemental Table 1** were added to the sample. After a 20 minute incubation, 1mL of fresh lysis buffer was added to each mixture. Following another 20 minute incubation, the sample was washed and run through a BD FACSCanto-II Flow Cytometer (339473, BD Biosciences) using the BD FACSDiva acquisition software. Stopping gate and events recorded were the same as that of the 4-colour assay. The 6-colour assay gating strategy is shown in **Supplemental figure 1A.**

*8-colour assay*

8-colour FACS analysis allowed us to investigate CD27 and CD57 expression in the same T-lymphocyte subsets as above. 100microL whole blood was incubated for 10 minutes with 5microL CCR7 antibody. Next, antibodies for CD8, CD3, PD-1, CD4, CD45, CD27 and CD57 were added together (antibody details given in **Supplemental Table 2**). 1mL of lysis buffer, prepared as above, was added and the mixture was vortexed, incubated for 20 minutes, then washed as for the 6-colour. Analysis was performed with an LSR II FACS machine and run to 20,000 CD3+ events. This is also illustrated in **Supplemental figure 1B.**

***Cardiac MRI***

In *CAPRI*, cMRI scans were obtained at 2-7 days and 12 ± 2 weeks post-reperfusion. In *Boag*, cMRI scans were obtained at 2-8 days following reperfusion.

*Image Acquisition*

Cardiac magnetic resonance (CMR) scans were obtained with a Siemens Avanto 1.5 Tesla MRI scanner, using a phased array body coil combined with a spine coil. All images were obtained during breath holding. Localiser images were acquired as well as axial black blood HASTE images to define anatomy. Cine images of the heart in 2, 3 and 4 chamber views were obtained using a steady state free precession pulse (SSFP) sequence (repetition time [TR]: set according to heart rate, image matrix 144x192, echo time (TE): 1.19ms, flip angle: 80°). T2 weighted STIR (short inversion time [TI] inversion recovery) images were then obtained in the same projections, using a black-blood segmented turbo spin echo technique (TR according to heart rate, TE 47ms, flip angle 180°, TI 140ms, image matrix 208x256). Further sequential end-diastolic STIR images were acquired along the short axis of the heart, covering the full extent of the left ventricle in parallel slices (each 8mm with 0mm gap). Corresponding short axis SSFP cine images were then obtained to allow quantification of chamber volumes and function. Intravenous Gadobutrol contrast (Gadovist, Bayer Schering Pharma AG, Berlin, Germany) was administered at a dose of 0.1mmol/kg, and after 10 minutes short axis end-diastolic LGE images (in corresponding locations to cine and STIR images) was obtained using an inversion recovery (IR) segmented gradient echo sequence (TR: according to heart rate, TE: 3.41ms, flip angle: 25°, image matrix: 196x256). The inversion time (TI) for LGE imaging was selected in order to null normal myocardium (giving it a dark appearance), and adjusted throughout acquisition (increased approximately every second slice) to maintain nulling.

*Image Analysis*

All analysis was performed using validated cardiac MRI analysis software (cvi42, Circle Cardiovascular Imaging Inc., Calgary, Canada). The analysis was performed by research fellows (A.M. and S.B.) and validated by cardiac imaging consultant (R.D), following formal training in CMR analysis and cvi42 software. In order to prevent bias, analysis was performed in batches, using anonymised images, which are subsequently linked back to the relevant clinical/FACS data. LV volumes and mass was determined using the short axis SSFP cine images, following determination of the longitudinal extent of the chamber by cross referencing with the 4 and 2 chamber images, as previously described and validated [40]. Epicardial and endocardial borders were traced automatically on each end-systolic and end-diastolic short axis cine frame with manual correction where necessary, allowing automated calculation of left ventricular mass, dimensions and ejection fraction (LVEF) (**Supplemental Figure 2**). In order to quantify infarct size and MVO, the short axis LGE images was used, all of which were taken at end diastole. Epicardial and endocardial borders were then traced on each LGE slice, and a reference region of normal myocardium identified using an automated method with manual correction where necessary. Areas of enhancement (infarction) was identified and quantified automatically using a signal intensity threshold of 5 standard deviations above normal remote myocardium, as previously described and validated [41]. Regions of hypoenhancement within the enhanced zone (microvascular obstruction – MVO), were identified and quantified using semi-automatic thresholding following manual border delineation of areas of interest, and included in the calculated infarct mass (**Supplemental Figure 3**).

***Mortality data***

The office of national statistics (ONS), a UK governmental body which records all deaths in the UK, provided mortality data. Mortality for 4874 consecutive STEMI patients receiving primary PCI was assessed as of 20/06/2016 and patients were identified by NHS number (a unique identification number). .

# Supplemental Tables

| **Antibody** | **Manufacturer** | **Catalogue Number** |
| --- | --- | --- |
| Brilliant Violet 421 anti-human CD197 (CCR7) | BioLegend | 353208 |
| BD Pharmingen FITC mouse anti-human CD8 | BD Biosciences | 555366 |
| BD Pharmingen PE mouse anti-human CD3 | BD Biosciences | 555333 |
| BD Horizon V500 mouse anti-human CD4 | BD Biosciences | 560768 |
| APC anti-human CX_3_CR1 | BioLegend | 341610 |
| CD45RA (L48) PE-Cy7 | BD Biosciences | 337186 |

**Supplemental table 1**. Antibodies used for the 6-colour flow cytometric assay with their respective catalogue number and manufacturer

| **Antibody** | **Manufacturer** | **Catalogue Number** |
| --- | --- | --- |
| Brilliant Violet 421 anti-human CD197 (CCR7) | BioLegend | 353208 |
| BD Pharmingen FITC mouse anti-human CD8 | BD Biosciences | 555366 |
| BD Pharmingen PE mouse anti-human CD3 | BD Biosciences | 555333 |
| BD Pharmingen APC mouse anti-human CD279 (PD1) | BD Biosciences | 558694 |
| BD Horizon V500 mouse anti-human CD4 | BD Biosciences | 560768 |
| APC anti-human CX_3_CR1 | BioLegend | 341610 |
| CD45RA (L48) PE-Cy7 | BD Biosciences | 337186 |
| BD Pharmingen Alexa Fluor mouse anti-human CD27 | BD Biosciences | 560611 |
| APC-Vio770 mouse anti-human CD57 | Milteney Biotech | 130-104-197 |

**Supplemental table 2**. Antibodies used for the 8-colour flow cytometric assay with their respective catalogue number and manufacturer

| **Variable** | **Lowest quartile of admission lymphocytes**  **(0.12-1.35 x10^9^/L)** | **2^nd^ quartile of admission**  **lymphocytes**  **(1.36 -1.9 x10^9^/L)** | **3^rd^ quartile of admission**  **lymphocytes**  **(1.91-2.68 x10^9^/L)** | **4^th^ quartile of admission**  **lymphocytes**  **(2.69-38.25 x10^9^/L)** | ***p* value** |
| --- | --- | --- | --- | --- | --- |
| Sample size, n (%) | 1224 (25.1) | 1225 (25.1) | 1219 (25.0) | 1206 (24.7) | - |
| Admission lymphocytes, x10^9^/L [median (IQR)] | 1.06 (0.87-1.22) | 1.62 (1.49-1.75) | 2.24 (2.09-2.43) | 3.41 (2.98-4.14) | - |
| Gender (male), n (%) | 891 (72.8) | 894 (73.0) | 838 (68.7) | 834 (69.2) | **0.028** |
| Age [years, mean (SD)] | 67.6 (12.9) | 62.5 (12.7) | 61.6 (12.5) | 59.8 (12.5) | **<0.001** |
| BMI [mean (SD)] | 26.7 (5.1) | 27.6 (5.2) | 28.0 (5.0) | 28.2 (5.2) | **<0.001** |
| Admission haemoglobin [mean (SD)] | 13.2 (1.9) | 13.7 (1.8) | 13.9 (1.7) | 14.2 (1.7) | **<0.001** |
| Admission creatinine [mean (SD)] | 96.0 (60.0) | 90.5 (45.6) | 90.0 (28.8) | 91.4 (33.3) | **0.001** |
| **Risk factors, n (%)** |  |  |  |  |  |
| **Smoking status** |  |  |  |  |  |
| Never smoked | 354 (31.9) | 329 (28.3) | 286 (24.7) | 214 (19.0) | **<0.001** |
| Ex-smoker | 437 (39.4) | 324 (27.9) | 283 (24.4) | 237 (21.0) | **<0.001** |
| Current smoker | 317 (28.6) | 510 (43.9) | 590 (50.1) | 676 (60.0) | **<0.001** |
| Family history of CAD | 456 (40.6) | 546 (47.2) | 532 (46.7) | 547 (48.6) | **0.001** |
| Hypertension | 569 (46.5) | 572 (46.7) | 503 (41.3) | 497 (41.2) | **0.003** |
| Diabetes Mellitus | 135 (11.2) | 145 (11.9) | 137 (11.4) | 1186 (13.6) | 0.270 |
| Hypercholesterolemia | 412 (33.7) | 440 (35.9) | 480 (39.4) | 429 (35.6) | **0.029** |
| **Medical history of CAD, n (%)** |  |  |  |  |  |
| Previous angina | 220 (18.3) | 228 (18.7) | 214 (17.7) | 218 (18.4) | 0.938 |
| Previous MI | 153 (12.7) | 142 (11.7) | 140 (11.6) | 127 (10.7) | 0.486 |
| Previous PCI | 85 (7.0) | 88 (7.2) | 63 (5.2) | 73 (6.1) | 0.160 |
| Previous CABG | 22 (1.8) | 30 (2.5) | 20 (1.6) | 16 (1.3) | 0.206 |
| **Clinical characteristics on admission** |  |  |  |  |  |
| Heart rate, bpm [median (IQR)] | 76 (63-90) | 75 (63-87) | 73 (63-87) | 72 (60-87) | 0.077 |
| Systolic BP, mmHg [median (IQR)] | 127 (109-148) | 130 (111-150) | 129 (110-148) | 126 (108-145) | **<0.001** |
| Interhospital transfer, n (%) | 421 (34.4) | 355 (29.0) | 338 (27.7) | 282 (23.4) | **<0.001** |
| Cardiogenic shock, n (%) | 57 (4.7) | 30 (2.5) | 39 (3.2) | 66 (5.5) | **<0.001** |
| LV ejection fraction [median (IQR)] | 40 (35-50) | 45 (35-55) | 45 (35-50) | 45 (35-53.75) | 0.233 |
| Total ischaemic time, min [median (IQR)] | 206 (137-324) | 182 (128-286) | 159 (114-255) | 137 (103-213) | 0.660 |
| Door to balloon, min [median (IQR)] | 26 (19-37) | 24 (18-34) | 23 (18-33) | 23 (18-32) | 0.425 |
| **Procedure details** |  |  |  |  |  |
| Radial access, n (%) | 927 (75.9) | 979 (80.0) | 971 (79.9) | 914 (75.8) | **0.008** |
| **Stenosed artery, n (%)** |  |  |  |  |  |
| Left main stem | 80 (6.5) | 74 (6.0) | 53 (4.3) | 66 (5.5) | 0.105 |
| Left anterior descending | 421 (34.4) | 374 (30.5) | 340 (27.9) | 330 (27.4) | **<0.001** |
| Left circumflex | 368 (30.1) | 321 (26.2) | 305 (25.0) | 292 (24.2) | **0.005** |
| Right coronary artery | 314 (25.7) | 306 (25.0) | 284 (23.3) | 288 (23.9) | 0.525 |
| No. vessels affected (0/1/2/3/4), n | 465/415/275/58/11 | 493/455/215/58/4 | 530/451/190/41/7 | 535/419/206/39/7 | **<0.001** |
| Number of stents [mean (SD)] | 1.52 (0.9) | 1.49 (0.9) | 1.53 (0.9) | 1.49 (0.9) | 0.552 |
| Infarct vessel caliber, mm [mean (SD)] | 3.50 (1.4) | 3.80 (10.8) | 3.51 (1.3) | 3.53 (1.2) | 0.485 |
| Total contrast used, ml [mean (SD)] | 156 (70) | 153 (154) | 144 (64) | 143 (66) | **0.003** |
| Thrombectomy used, n (%) | 577 (47.3) | 623 (51.0) | 643 (52.9) | 661 (54.9) | **0.001** |
| GPI IIb/IIIa used, n (%) | 840 (68.7) | 884 (72.2) | 907 (74.4) | 876 (72.6) | 0.167 |
| **TIMI flow pre-PCI in culprit artery, n (%)** |  |  |  |  |  |
| 0 | 831 (70.0) | 832 (70.4) | 853 (72.2) | 889 (76.0) | **0.004** |
| 1 | 69 (5.8) | 59 (5.0) | 52 (4.4) | 59 (5.0) | 0.481 |
| 2 | 114 (9.6) | 115 (9.7) | 118 (10.0) | 101 (8.6) | 0698 |
| 3 | 173 (14.6) | 175 (14.8) | 158 (13.4) | 120 (10.3) | **0.004** |
| **TIMI flow post-PCI in culprit artery, n (%)** |  |  |  |  |  |
| 0 | 33 (2.8) | 23 (2.0) | 29 (2.5) | 22 (1.9) | 0.367 |
| 1 | 16 (1.4) | 15 (1.3) | 6 (0.5) | 9 (0.8) | 0.111 |
| 2 | 68 (5.8) | 46 (4.0) | 34 (2.9) | 35 (3.0) | **0.001** |
| 3 | 1047 (89.9) | 1080 (92.8) | 1086 (94.0) | 1103 (94.4) | **<0.001** |
| **Outcome** |  |  |  |  |  |
| Days alive [mean (SD)] | 1757 (860) | 1829 (799) | 1893 (812) | 1882 (828) | **<0.001** |
| Intrahospital death, n (%) | 48 (3.9) | 33 (2.7) | 29 (2.4) | 52 (4.3) | **0.019** |

**Supplemental table 3 –** demographics for the cohort of 4874 consecutive STEMI patients, analysed retrospectively.

| Variable | **p value** | **Hazard ratio** | **Hazard ratio 95% CI** |
| --- | --- | --- | --- |
| Anterior MI | 0.001 | 0.778 | 0.672-0.902 |
| Creatinine per 100mmol | <0.001 | 1.379 | 1.271-1.495 |
| Haemoglobin | <0.001 | 0.899 | 0.863-0.937 |
| Heart rate per 10bpm | <0.001 | 1.046 | 1.032-1.060 |
| Current smoker | <0.001 | 0.718 | 0.608-0.848 |
| Previous MI | <0.001 | 0.606 | 0.507-0.724 |
| Diabetes | <0.001 | 0.637 | 0.525-0.773 |
| **Lymphocyte quartile vs 4^th^ quartile** |  |  |  |
| Lowest quartile | 0.004 | 1.371 | 1.109-1.695 |
| 2^nd^ Quartile | 0.486 | 1.083 | 0.865-1.356 |
| 3^rd^ Quartile | 0.881 | 0.983 | 0.781-1.236 |
| Age per decade | <0.001 | 2.090 | 1.937-2.256 |

**Supplemental table 4** – Variables includes in the final multivariate analysis model of 4874 consecutive STEMI patients undergoing pPCI. Variables included in the initial analysis were anterior myocardial infarction, creatinine, haemoglobin, heart rate on presentation, current smoking, previous myocardial infarction, diabetes mellitus, lymphocyte quartile, age and gender. Gender was excluded from the final model as it was not significantly associated with survival.

|  | CMV Positive (n=58) | CMV Negative (n=43) | p value |
| --- | --- | --- | --- |
| Age (years) | 64.7 ± 1.3 | 58.0 ± 1.6 | 0.0027 |
| Male | 45 (77.6) | 39 (90.7) | 0.11 |
| BMI (kg/m^2^) | 27.6 ± 0.6 | 27.0 ± 1.5 | 0.87 |
| Past CAD | 2 (3.4) | 2 (4.7) | >0.99 |
| Family history of CAD | 28 (48.3) | 15 (34.9) | 0.22 |
| Hypertension | 15 (25.9) | 9 (20.9) | 0.64 |
| Hypercholesterolaemia | 12 (20.7) | 10 (23.3) | 0.81 |
| Diabetes mellitus | 5 (8.6) | 3 (7.0) | >0.99 |
| Current smoker | 18 (31.0) | 20 (46.5) | 0.15 |
| Anterior MI | 21 (36.2) | 20 (46.5) | 0.31 |
| Peak troponin (ng/L) | 4481 ± 400 | 4807 ± 536 | 0.77 |
| **Pre-admission medication** |  |  |  |
| Aspirin | 5 (8.6) | 3 (7.0) | >0.99 |
| Β-blocker | 1 (1.7) | 2 (4.7) | 0.57 |
| ACE-inhibitor/ARB | 7 (12.1) | 4 (9.3) | 0.75 |
| Statin | 13 (22.4) | 6 (14.0) | 0.32 |
| Calcium channel blocker | 6 (10.3) | 4 (9.3) | >0.99 |
| **Procedure details** |  |  |  |
| Onset to balloon time (min) | 181 ± 11 | 171 ± 14 | 0.33 |
| >1 vessel treated | 5 (8.6) | 4 (9.3) | >0.99 |
| Flow pre-PCI (TIMI 0/1/2/3) | 49/9/0/0 | 40/3/0/0 | 0.23 |
| Flor post-PCI (TIMI 0/1/2/3) | 0/2/0/56 | 0/0/0/43 | 0.51 |

**Supplemental table 5 -** Demographic characteristics of all patients who underwent an acute post-infarct cardiac MRI, split by CMV serostatus (n=101). Continuous variables are presented as mean ±SEM. Discrete variables are presented as count (%). p values were determined with the Mann-Whitney U test for continuous variables and Fischer’s exact test for discrete variables. Statistical significance defined as p<0.05.

|  | **STEMI (n=19)** | **Controls (n=10)** |
| --- | --- | --- |
| Age (years) | 60.6 ± 2.8 | 29.1 ± 2.8 |
| Male | 15 (78.9) | 5 (50) |
| BMI (kg/m^2^) | 27.4 ± 0.9 | - |
| Past CAD | 2 (10.5) | - |
| Family history of CAD | 8 (42.1) | - |
| Hypertension | 6 (31.6) | - |
| Hypercholesterolaemia | 1 (5.3) | - |
| Diabetes mellitus | 1 (5.3) | - |
| Current smoker | 9 (47.4) | - |
| Anterior MI | 12 (63.2) | - |
| Peak troponin (ng/L) | 3140 ± 621 | - |
| **Pre-admission medication** |  |  |
| Aspirin | 1 (5.3) | - |
| Β-blocker | 1 (5.3) | - |
| ACE-inhibitor/ARB | 2 (10.5) | - |
| Statin | 2 (10.5) | - |
| Calcium channel blocker | 3 (15.8) | - |
| **Procedure details** |  |  |
| Onset to balloon time (min) | 130.6 ± 15.1 | - |
| >1 vessel treated | 5 (26.3) | - |
| Flow pre-PCI (TIMI 0/1/2/3) | 19/0/0/0 | - |
| Flor post-PCI (TIMI 0/1/2/3) | 0/0/0/19 | - |

**Supplemental table 6 -** Demographic characteristics of patients whose samples were used to investigate associations between CX_3_CR1, CD27 and CD57. Also used to compare CX_3_CR1 expression at 24 hours between STEMI patients and healthy controls (no relevant past medical history, and on no relevant medications). Split into STEMI and controls (n=29). Continuous variables are presented as mean ± SEM. Discrete variables are presented as count (%).

|  | Coefficient | 95% Confidence interval for coefficient | p value |
| --- | --- | --- | --- |
| Age (years) | -0.456 | -1.224 – 0.311 | 0.237 |
| Current smoker | 2.021 | -8.228 – 12.271 | 0.692 |
| History of hypercholesterolemia | -0.634 | -18.940 – 17.671 | 0.945 |
| Statins as premedication | 0.001 | -0.001 – 0.003 | 0.369 |
| Peak troponin (ng/mL) | 13.554 | -1.039 – 28.147 | 0.068 |
| CMV IgG serostatus | 5.419 | -9.688 – 20.526 | 0.473 |
| Anterior infarct | -7.065 | -20.687 – 6.557 | 0.301 |

**Supplemental table 7**- Full list of variables entered into multivariate linear regression model for change in end-systolic volume over 12 weeks. Adjusted coefficients with 95% confidence intervals are shown for all covariates, along with p values for coefficient. n=52 with no missing values.

#
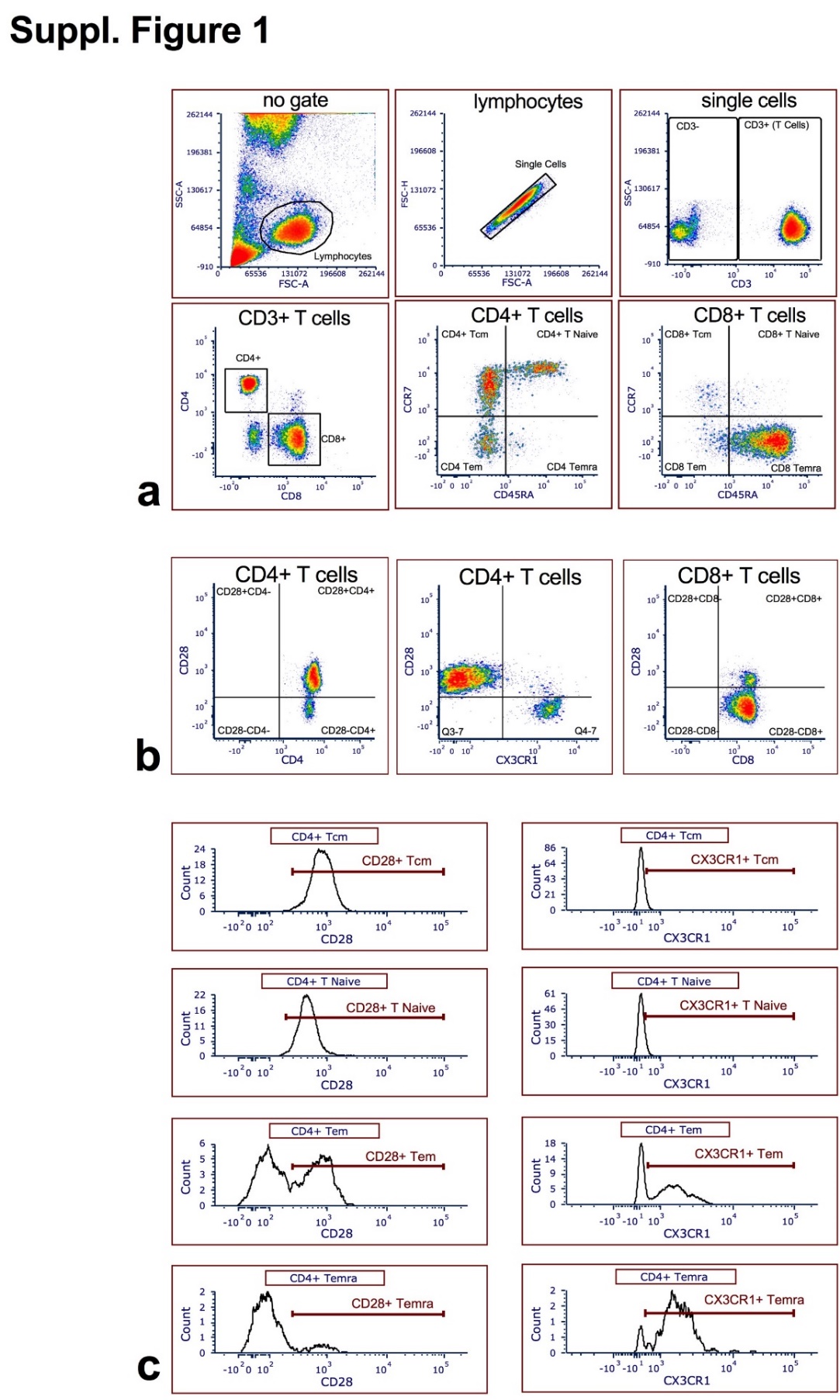
Supplemental Figures

**Supplemental Figure 1** **A:** Flow cytometry gating strategy in the 6-colour assay. Lymphocytes were identified and split first by CD3 expression. CD3^+^ T-cells were split into CD4^+^ and CD8^+^, and these were then split into naïve, central memory, effector memory and terminally differentiated effector memory cells by expression of CCR7 and CD45RA **B:** In the 8-colour assay, CD28 was measured in CD4^+^ and CD8^+^ T-cells, and co-expression with CX_3_CR1 was quantified. **C:** Histograms representing CD28 and CX_3_CR1 expression in all subsets of CD4^+^ T-cells.

**Supplemental Figure 2 -** LV mass and volume assessment by CMR **A**: Basal ventricular short axis slice at end diastole showing endocardial border (red), epicardial border (green) **B**: Corresponding long axis reference image in both 2 and 4 chamber views, showing the slice position (highlighted in yellow). **C+D**: Equivalent short axis slice and long axis reference images at end systole.

**Supplemental Figure 3 -** Analysis of LGE images for infarct size and MVO quantification. **A+B:** Short axis LGE image showing inferior infarct. **A:** Raw image without analysis, in which normal myocardium appears dark and infarct zone shows enhanced (white) appearance **B:** Corresponding analysed image showing myocardial borders (red: endocardial, green: epicardial) as well as normal myocardium reference area (blue border) and region of enhancement (infarct, yellow shading). **C+D**: Short axis LGE images showing anteroseptal infarct with extensive MVO **C:** Raw unanalysed image in which dark core of MVO can be clearly seen within hyperenhanced infarct. **D** Corresponding analysed image shows all contours and analysis, with infarct area shown in yellow and MVO shaded orange.


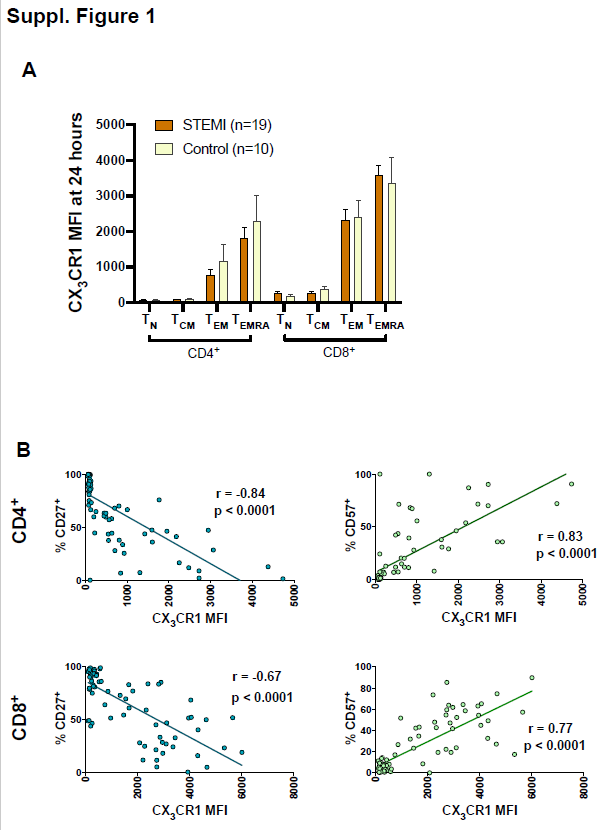


**Supplemental figure 4 – A:** CX_3_CR1 expression at 24 hours in STEMI *vs* controls, n=28. There was no significant difference observed in CX_3_CR1 expression in any subset, as assessed by unpaired t-test with a significance threshold of p<0.05. **B:** Correlation between expression of CX_3_CR1 and expression of CD27 (left) and CD57 (right) in CD4^+^ (top) and CD8^+^ (bottom) T-lymphocytes. 19 patients with each subset (T_N_, T_CM_, T_EM_, T_EMRA_) plotted for each patient (n=78). Correlation with Spearman’s test.
